# Laparoscopic Abdominoperineal Resection of Undifferentiated Spindle Cell Sarcomas of the Rectum with Lymph Node Metastases: A Rare Case Report

**DOI:** 10.70352/scrj.cr.24-0049

**Published:** 2025-01-31

**Authors:** Ryo Shibayama, Yutaka Hanaoka, Yutaka Takazawa

**Affiliations:** 1 Department of Gastroenterological Surgery, Toranomon Hospital, Tokyo, Japan; 2 Department of Pathology, Toranomon Hospital, Tokyo, Japan

**Keywords:** undifferentiated sarcoma, lymph node metastasis, spindle cell, undifferentiated pleomorphic sarcoma, malignant fibrous histiocytoma, abdominoperineal resection, aged people, laparoscopic surgery

## Abstract

**INTRODUCTION:**

Undifferentiated sarcomas of the gastrointestinal tract are rare and have poor prognoses, especially those with lymph node metastases. There is no consensus on the treatment plan. While there are reports on undifferentiated pleomorphic sarcomas of the rectum, no reports on undifferentiated rectal spindle sarcomas with lymph node metastases have been presented previously.

**CASE PRESENTATION:**

We report a case of a 97-year-old woman referred to our hospital with anal pain. Imaging findings indicated multiple tumors in the rectum below the peritoneal reflection protruding from the anus and two enlarged pararectal lymph nodes. Laparoscopic abdominoperineal resection of the rectal sarcomas with lymph node metastasis was performed to alleviate the pain with uneventful postoperative courses. The immunostaining did not reveal a trend of tumor cell differentiation. The tumor was diagnosed as undifferentiated spindle cell sarcoma based on histopathological findings. Because of advanced age, the patient is followed up on an outpatient basis without additional postoperative treatment.

**CONCLUSION:**

The prognosis of undifferentiated sarcomas is poor. While radical resection is the primary treatment, the efficacy of preoperative radiation therapy, cytotoxic chemotherapy, and immune checkpoint inhibitors has been investigated recently. Accumulating cases of this disease is important to determine treatment plans, and this report is valuable in this regard.

## Abbreviations

CT
: computed tomography
FDG
: fluorodeoxyglucose
GI
: gastrointestinal
MFH
: malignant fibrous histiocytoma
MRI
: magnetic resonance imaging
PET
: positron emission tomography
WHO
: World Health Organization
UPS
: undifferentiated pleomorphic sarcomas

## INTRODUCTION

The recent World Health Organization (WHO) classification system subdivides undifferentiated sarcomas into undifferentiated round, spindle, pleomorphic, and epithelioid cells, as well as undifferentiated sarcomas. According to the 2017 WHO classification system, a group of tumors comprising spindle-shaped cells are considered malignant tumors.^1)^ Previous systems used different classifications such as malignant fibrous histiocytoma (MFH).^2)^ Undifferentiated sarcomas can occur in any body part, most commonly in the extremities.^3)^ These tumors are rare and have a poor prognosis, with no consensus on the treatment plan. They rarely occur in the gastrointestinal (GI) tract. Although undifferentiated pleomorphic sarcomas (UPS) in the rectum have been reported previously,^4)^ no reports of undifferentiated spindle sarcoma in the rectum have been presented. We report a case of laparoscopic abdominoperineal resection of undifferentiated spindle cell sarcomas of the rectum with lymph node metastases and conducted a literature review. MFH was first described as malignant histiocytoma and fibrous xanthoma by Ozzello et al. in 1963 and as malignant fibrous xanthoma the following year by O’Brien and Stout in the same group.^5,6)^ In 1978, the clinicopathologic features of 200 cases of MFH were analyzed and the concept of MFH was established. At that time, MFH was thought to be a malignant tumor derived from pleomorphic spindle cells that could differentiate into histiocytes and fibroblasts.^3)^ However, advances in research have proposed that the histogenesis of MFH is undifferentiated mesenchymal cells. Therefore, the concept of MFH was removed from the WHO classification of diseases in 2002 and 2013. The recent WHO classification system subdivides undifferentiated sarcomas into undifferentiated round, spindle, pleomorphic, and epithelioid sarcomas.^1)^

## CASE PRESENTATION

**Fig. 1:**
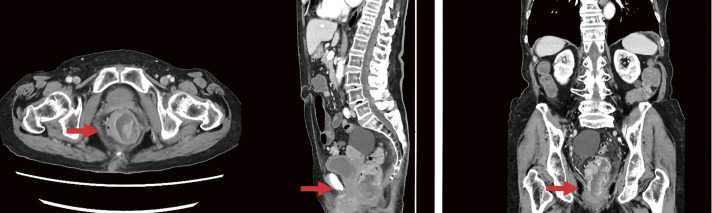
Computed tomography images. Multiple tumors on the left wall of the rectum below the peritoneal reflection protruding from the anus and two enlarged pararectal lymph nodes were observed, while no obvious distant metastasis was found.

A 97-year-old woman was referred to our hospital with anal pain. The patient underwent trans-anal resection of a tumor protruding from the rectum and anus by her previous surgeon 6 months before he was admitted to our hospital. Pathological results indicated a sarcoma with positive margins; however, the patient was monitored without any additional treatment because of advanced age. The patient had no other major illnesses. Computed tomography (CT) revealed multiple tumors on the left wall of the rectum below the peritoneal reflection protruding from the anus, two enlarged pararectal lymph nodes, and no obvious distant metastasis (**Fig. 1**). Laparoscopic abdominoperineal resection was performed to relieve severe pain.

**Fig. 2:**
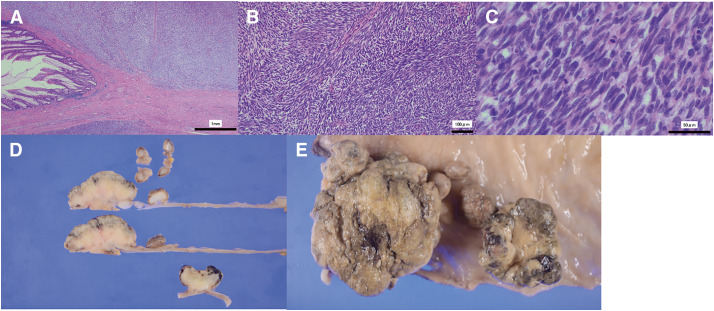
Histopathological findings. Atypical short spindle cells proliferated in a storiform or fascicular pattern within a myxoid stroma. The tumor cells showed mild pleomorphism, and multinucleated giant or epithelioid cells were evident. Venous and lymph vessel permeation was noted. Immunohistochemically, the tumors were partially positive for S-100 protein, muscle-specific actin, CD10, cyclinD1, PgR, and MIC2. KIT, DOG1, CD34, desmin, h-Caldesmon, αSMA, GFAP, HMB45, and MelanA tests were all negative. No loss of H3K27me3 was observed. The Ki-67 index was approximately 80%. The tumor was diagnosed as undifferentiated spindle cell sarcoma. (**A**) HE, ×40. (**B**) HE, ×200. (**C**) HE, ×800. (**D** and **E**) Macroscopic findings of the resected specimen.

Surgery was initiated with the patient in the lithotomy position under general and epidural anesthesia. A 12-mm, two 5-mm, and two 12-mm camera ports were inserted into the umbilicus, either side of the upper abdomen and lower abdomen. Intra-abdominal observation revealed no liver metastases or peritoneal dissemination. The inferior mesenteric artery was dissected at the root, and regional lymph node dissection was performed. The fascia propria of the rectum was dissected with a layer of nerve-sparing total mesenteric excision to the pelvic floor. The pelvic floor muscles were incised to expose the sciatico-rectal fat, anteriorly rounded and detached from the dorsal side of the vagina. Perineal manipulation was subsequently performed to remove the specimen. The surgical duration was 3 hours and 50 minutes. Macroscopically, multiple polypoid tumors sized up to 62 × 54 × 25 mm were observed in the Rb/P region, mainly located in the submucosal layer with an ulcerative surface, and the largest one appeared to originate from the muscularis propria. No sphincter or adventitia involvement was observed. Histopathologically, atypical short spindle cells proliferated in a storiform or fascicular pattern within a myxoid stroma. The tumor cells showed mild pleomorphism, and multinucleated giant or epithelioid cells were evident. Venous and lymph vessel permeation was noted. Immunohistochemically, the tumors were partially positive for S-100 protein, muscle-specific actin, CD10, cyclinD1, PgR, and MIC2. On the other hand, KIT, DOG1, CD34, desmin, h-Caldesmon, αSMA, GFAP, HMB45, and MelanA tests were all negative. No loss of H3K27me3 was observed. The Ki-67 index was approximately 80%. The tumor was diagnosed as undifferentiated spindle cell sarcoma (**Fig. 2**). The postoperative course was uneventful, and the patient was discharged on postoperative day 11. Given the advanced age, chemotherapy was not administered, and she has been followed on an outpatient basis for 2 years postoperatively without any recurrences.

## DISCUSSION

Primary colorectal sarcomas are rare, accounting for only 0.1% of all colorectal malignancies, and may occur in the colon (70.7%) or rectum (25.4%).^7)^ The prognosis is poor, with a reported 5-year survival rate of 43.8%.^7)^ Radical resection including lymph node dissection is often performed. Of patients undergoing lymph node dissection for primary colorectal sarcoma, only 10% are positive for lymph node metastasis.^8)^ Leiomyosarcomas are the most common type, accounting for >90% of primary colorectal sarcomas. Other common benign mesenchymal tumors of the GI tract include lipomas, leiomyomas, vascular lesions, and nerve sheath tumors. In a study examining 658 sarcomas in adults, 22 (3.3%) were secondary sarcomas developed approximately 10 years (3–45 years) after radiation therapy.^8)^ There have been a few reports of radiation-induced sarcomas occurring in the GI tract.^9–11)^ GI sarcomas may be detected incidentally or present with abdominal pain, GI bleeding, anemia, or an abdominal mass. Magnetic resonance imaging (MRI) is the best-suited method for evaluating soft tissue tumors for its excellent tissue resolution and the characteristic findings have particular diagnostic value in cystic diseases. Contrast-enhanced MRI may be useful in differentiating cysts from hemangiomas and enhancing tumors from hemangiomas. The lungs are the most common site of metastasis.^12,13)^ Therefore, chest CT is recommended for evaluating pulmonary metastatic diseases. Positron emission tomography (PET) is not recommended for diagnosing sarcomas because it cannot effectively differentiate soft tissue tumors from moderate sarcomas. In addition, myxoid liposarcoma and synovial sarcoma metastases may have low fluorodeoxyglucose (FDG) avidity, resulting in a higher rate of false-negative results compared to MRI.^14)^ However, PET may be used to search for metastases in the lymph nodes and other organs. Pathological examination of the resected specimen is necessary to obtain a definitive diagnosis. However, diagnoses in many cases are difficult because of various histological types and the rarity of the disease. A pathological diagnosis is generally made based on histomorphology and clinical or radiological images, while immunohistochemical staining and genetic testing are used as necessary. According to the WHO, most soft tissue neoplasms are classified according to their presumed tissues of origin. When tissue origin is uncertain, sarcomas are designated based on their structural patterns, and immunostaining is used to determine the origin.

Kodera et al. summarized 29 cases of UPS/MFH occurring in the colorectum found in PubMed.^4)^ No patients received preoperative treatment, and only a few received adjuvant chemotherapy or radiation therapy. In almost all cases, patients died during the early postoperative period. Preoperative radiation therapy for retroperitoneal sarcoma has been reported to reduce local recurrence and improve prognosis.^15,16)^ Few cases of UPS have been reported, and the evidence for preoperative treatment remains unclear. However, radiation therapy is important to shrink the tumor and provide local control. Because sarcomas are at high risk of local recurrence, preoperative chemotherapy may have a negative prognostic impact. Cytotoxic therapy consisting of doxorubicin and ifosfamide is recommended for unresectable sarcomas with distant metastases. The expression of genes related to the immune response is known to be high in UPS, and treatment with the combination of nivolumab and ipilimumab or with pembrolizumab has been reported to be effective.^17,18)^

A diagnosis could not be made based on histomorphology or immunostaining in this patient. Therefore, the tumor was classified as undifferentiated spindle cell sarcoma according to the WHO classification. Although the tumor was morphologically different from UPS, we believe the treatment strategy would be similar. In the present case, the patient was of advanced age, and the curative value was considered less important. Therefore, no preoperative treatment was provided, and surgery was not planned initially. However, to relieve her anal pain, we decided to perform surgery. In the future, these types of tumors should be treated with preoperative radiation therapy and immune checkpoint inhibitors combined with more curative resection. Radiation is one of the treatment options if only for pain relief. In this case, resection was the better option because the patient had no recurrence in the observation period of 2 years after the surgery. Due to the rarity and poor prognosis of undifferentiated spindle cell sarcomas, this report is of significant value.

## ACKNOWLEDGMENTS

We would like to thank Editage (www.editage.jp) for English language editing.

## DECLARATIONS

### Funding

The authors received no financial support for the research, authorship, and/or publication of this article.

### Authors’ contributions

The final manuscript was read and approved by all authors.

All authors agree to be responsible for all aspects of the study.

Ryo Shibayama: Paper writing and conception.

Yutaka Hanaoka: Guidance for writing papers.

Yutaka Takazawa: Provision of histopathology images.

### Availability of data and materials

Data that support the findings of this study are available from the corresponding author upon reasonable request.

### Ethical approval and consent to participate

This work does not require ethical considerations or approval. A written informed consent was obtained from the patient.

### Consent for publication

A written informed consent for publication was obtained from the patient.

### Conflict of interest

The authors declare no conflict of interest for this article.
